# Necrobiosis Lipoidica Mimicking Chronic Infectious Ulceration: A Case Report and Literature Review

**DOI:** 10.7759/cureus.114306

**Published:** 2026-08-10

**Authors:** Kylee Arthurs, Brianna M Tischner, Nathalie Ruiz, Michael Higginbotham, Juan Socas

**Affiliations:** 1 General Surgery, HCA Florida Brandon Hospital, Brandon, USA; 2 Medicine, HCA Florida Orange Park Hospital, Orange Park, USA; 3 Pathology, HCA Florida Brandon Hospital, Brandon, USA; 4 Plastic Surgery, HCA Florida Brandon Hospital, Brandon, USA

**Keywords:** autoimmune, general dermatology, necrobiosis lipoidica, pathergy, plastic surgergy, wide surgical resection, wound care

## Abstract

Necrobiosis lipoidica (NL) is a rare, granulating autoimmune disease that can be quite difficult to diagnose. It has a higher incidence in diabetic patients than in the general population. Diagnosis often depends upon both clinical and histopathologic examinations. It has a frequent association with diabetes, metabolic syndrome (obesity, hypertension, high cholesterol), and thyroid diseases. This is the case of a 50-year-old male patient who developed ulcerative skin lesions that were refractory to antibiotic therapy. He underwent antibiotic treatments for about two years, as well as multiple surgical debridement with skin grafting. However, the lesions continued to recur. Finally, after two years, a diagnosis of NL was made. He was subsequently prescribed steroids and immunologics, which improved his symptoms significantly. NL treatment is determined by whether the lesions are ulcerative or not. Typically, management will begin with topical corticosteroids and advance to systemic treatments as indicated. Surgical interventions are generally not recommended, as this disease is known to have pathergy. NL is a rare disease that is difficult to diagnose. It can be mistaken for an infectious etiology, like in our case. Due to the difficulty in diagnosing the disease, treatment can be delayed and, in turn, result in unintended harm to the patient with unnecessary procedures. In the future, there should be further investigation into the pathogenesis of this disease and advancement of diagnostic tools for earlier, accurate diagnosis.

## Introduction

Necrobiosis lipoidica (NL) is a rare autoimmune condition with cutaneous manifestations. It was first described in 1929 as necrobiosis lipoidica diabeticorum. However, by 1948, there were numerous cases reported among nondiabetic patients; thus, it was renamed simply as necrobiosis lipoidica. The risk factors for developing this disease seem to be associated with poorly controlled diabetes, hypertension, metabolic syndrome, or even poorly controlled venous insufficiency [1]. The pathophysiology of this disease is poorly understood, and several theories have been suggested. For example, it is suggested that NL develops as vascular disease precipitated by immunologic dysfunction. One suggestion is a connection to IgM deposition within the dermoepidermal junction, causing microvascular changes resulting in collagen degeneration. Another suggestion in relation to diabetes mellitus-associated NL is that there is abnormal collagen deposition that will increase collagen cross-linking and cause thickening of the basement membrane, which is commonly seen in NL. Additionally, there are theories regarding impaired neutrophil migration resulting in granulomas, as well as tumor necrosis factor (TNF)-alpha-mediated granuloma formation [1]. However, currently there is no clearly identified causal relationship. 

The mean age of onset of NL for nondiabetic patients is 52 versus 45 for those with diabetes [2]. As is the case with many autoimmune diseases, there is a higher incidence of NL in women compared to men, as high as 8:1 in some studies [2]. A complication of the cutaneous lesions of NL is ulceration, which can lead to skin necrosis and the need for surgical intervention. The most frequently cited surgical recommendations are from case series from Stanford University Medical Center performed in the 1970s, which described remission in patients who had debridement to deep fascia or periosteum, followed by eventual split-thickness skin grafts [3]. However, care should be taken with the use of surgical intervention, as NL has a tendency for pathergy or Koebnerization, in which lesions can be made worse by the trauma of surgery [2].

This case report discusses a 50-year-old male patient with a past medical history of hyperlipidemia, but who is otherwise healthy, who had recurrent ulcerations to the lower extremity over two years before finally achieving an accurate diagnosis. This case report will discuss a patient who had a prolonged delay in diagnosis, repeated unsuccessful surgeries, followed by an appropriate histopathologic diagnosis and successful medical management. 

## Case presentation

A 50-year-old male patient with a past medical history significant for hyperlipidemia presented with chronic, nonhealing right lower extremity ulcers. These ulcers had been ongoing for two years. His first episode began after a vacation where he spent time submerged in a tropical body of water. The condition initially started as erythematous plaques and nodules that would open and drain seropurulent fluid (Figure 1). Over these two years, he had been worked up by multiple specialists and had a debridement with skin grafting, all at outside facilities. At one point during his two-year-long workup, he was told he had a positive culture for *Pseudomonas aeruginosa* and a presumed positive for acid-fast bacilli. At that time, the discussion with his care team was a possible atypical mycobacterium infection. Due to this infectious concern, he was treated with a prolonged course of intravenous antibiotics. 

**Figure 1:**
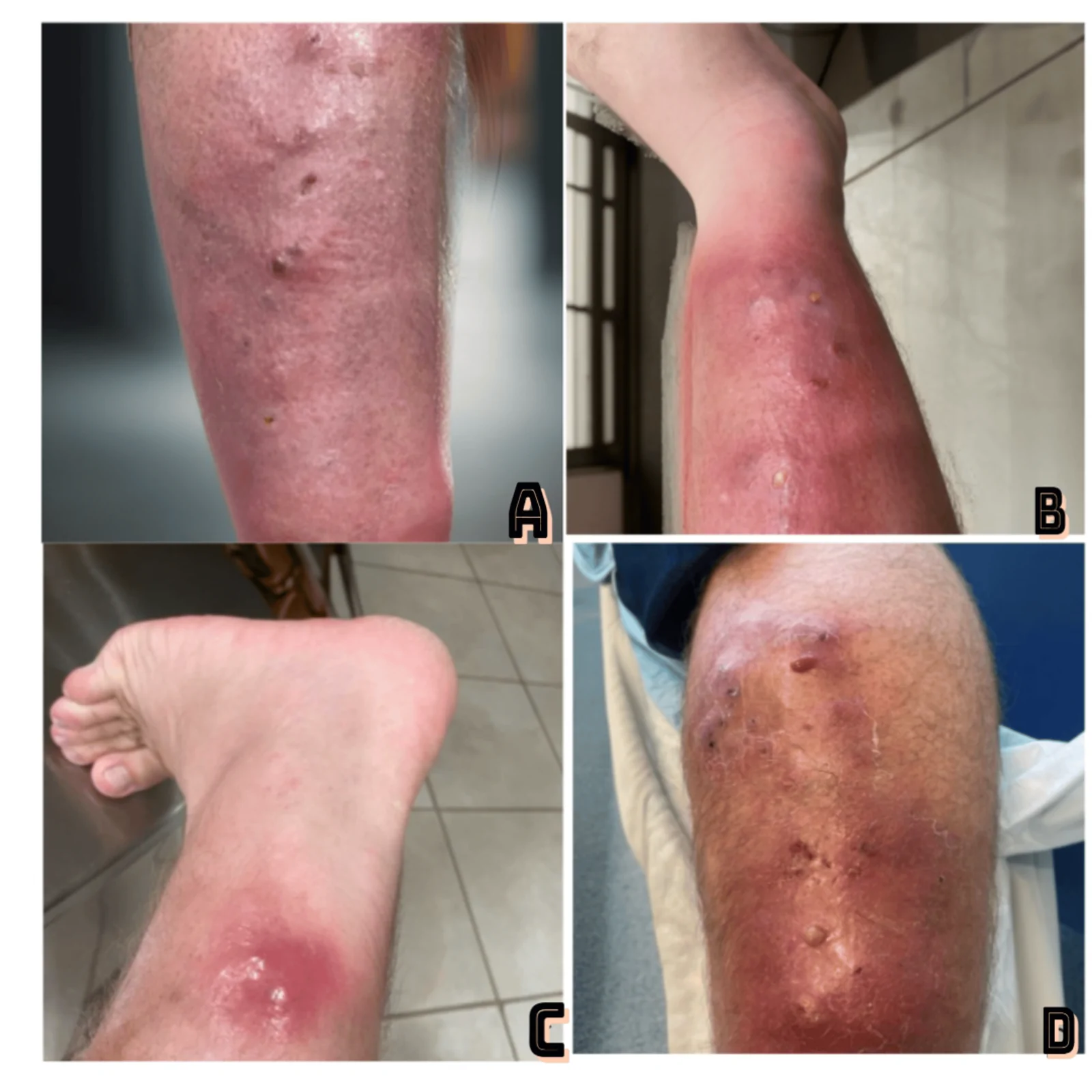
initial presentation of wounds, prior to treatment (A) Anterior tibial lesions of erythematous nodules with intermittent areas of ulceration, oriented superior to inferior in the picture. (B) Additional anterior tibial lesions on the distal aspect of the limb, with erythematous nodules that show evidence of seropurulent drainage. (C) Large erythematous atrophic nodule on the posterior lower limb just proximal to the lateral malleolus. (D) Additional atrophic nodules along the posterior lower extremity just inferior to the popliteal space seen at the superior aspect of the picture, with areas of ulceration and intermittent areas of active drainage

However, other working diagnoses at that time included pyoderma gangrenosum, granuloma annulare, and cutaneous sarcoidosis. These were ruled out with multiple specimen samples sent for pathology, which did not show the characteristics notable to those disease states. 

After these treatment options failed, and toward the end of the two-year course, he presented to our wound care clinic for evaluation. With our initial physical exam, we noted ulcerative lesions with necrosis on the posterior calf in the area of a prior skin graft (Figure 2). Surgical debridement was recommended. During our index surgery in late fall 2024, cultures and tissue were sent, which subsequently returned negative for infectious etiology. However, pathology was positive for coagulative necrosis. At that time, it was assumed the samples were falsely negative, and local wound care with antibiotic treatment was continued. 

**Figure 2:**
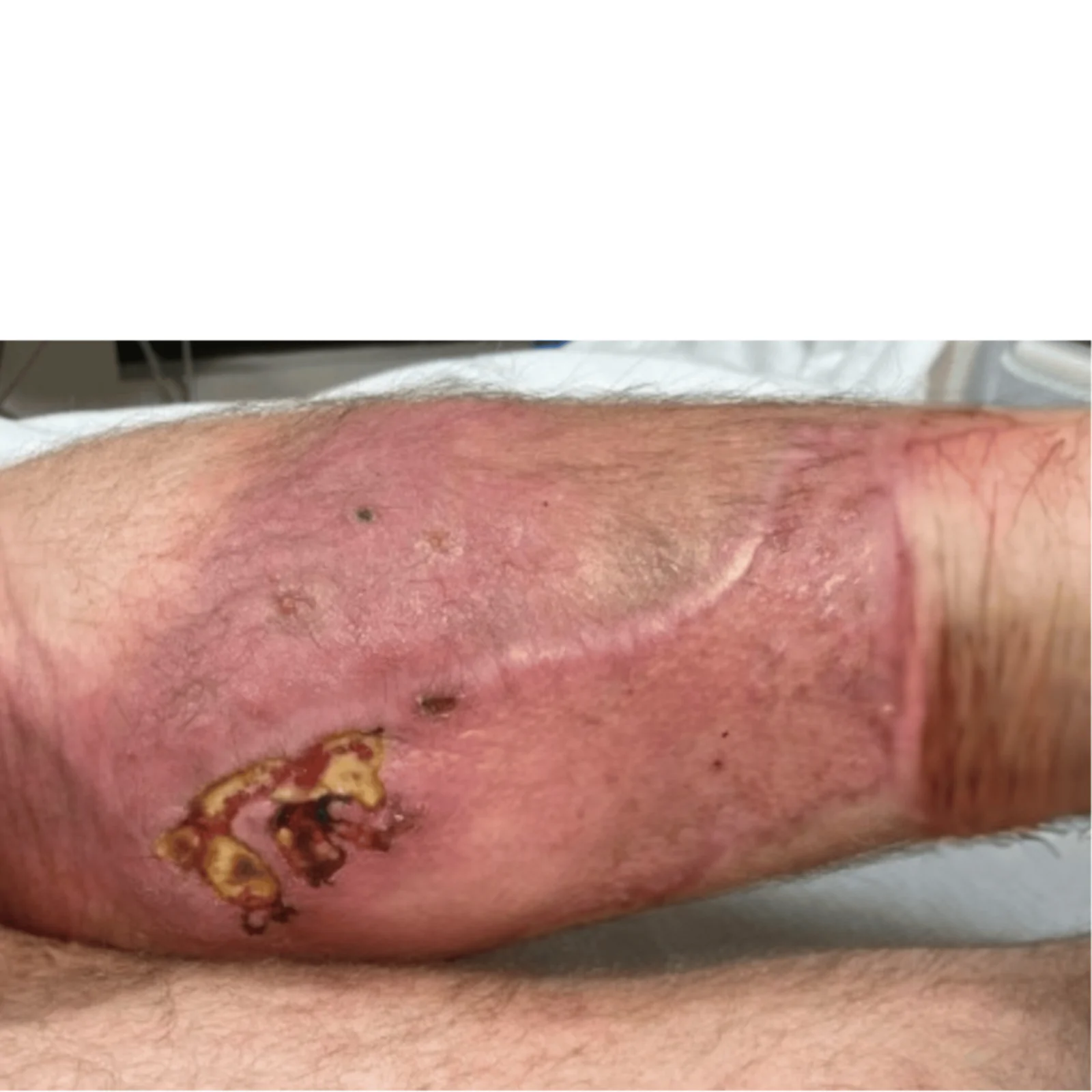
Recurrent ulceration within area of prior skin grafting Posterior lower limb, just below the popliteal space, which is located on the left of the picture, at the superolateral aspect of the prior skin graft, has an ulcerative lesion with evidence of necrosis

Unfortunately, within a few weeks, he was noted to have new draining nodules on the anterior tibia, within an area that had previously been debrided and skin grafted (Figure 3). These wounds were treated with local wound care; however, by midwinter, additional necrosis was noted. He returned to the operating room for debridement and preparation for skin allograft. During this procedure, the affected areas were debrided down to muscle fascia with allograft placement. 

**Figure 3:**
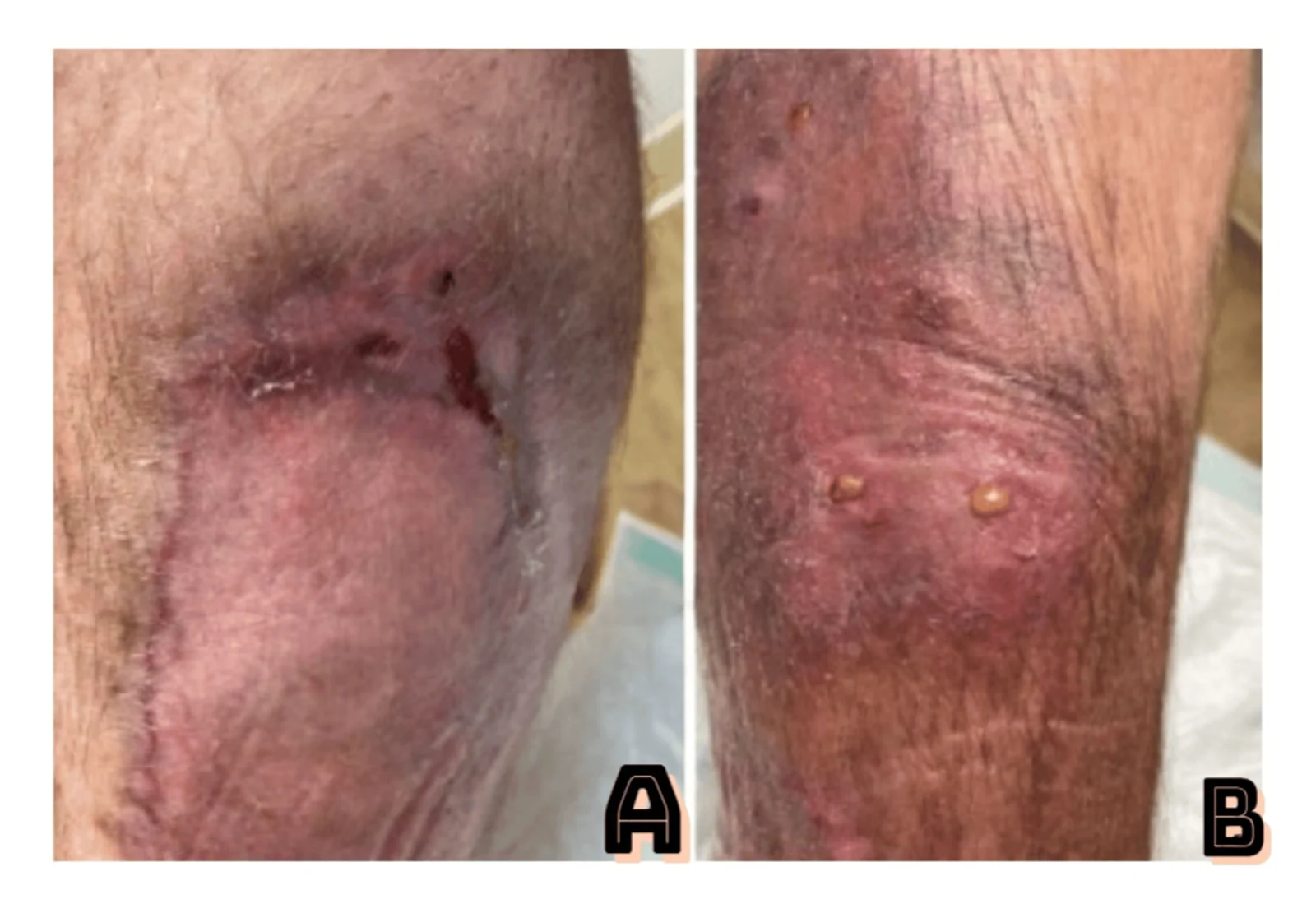
Healing posterior calf wound after debridement (L), recurrent nodules on the anterior tibia (R) (A) Anterior tibia with area of healed skin graft with evidence of ulceration at the superior aspect. (B) Same area on the anterior tibia with healed ulcer but now with recurrent nodular lesions with ulceration and active drainage

After 5-7 days of allograft preparation, the patient returned to the OR for autografting with a donor site of anteromedial right thigh. Initially, both donor and recipient sites were well-healed. Unfortunately, by early springtime 2025, there were recurrent necrotic ulcers forming in the areas of skin grafting (Figure 4). These ulcers were subsequently treated with local wound care using a wet-to-dry method. Figure 5 shows the wound progression with this method. 

**Figure 4:**
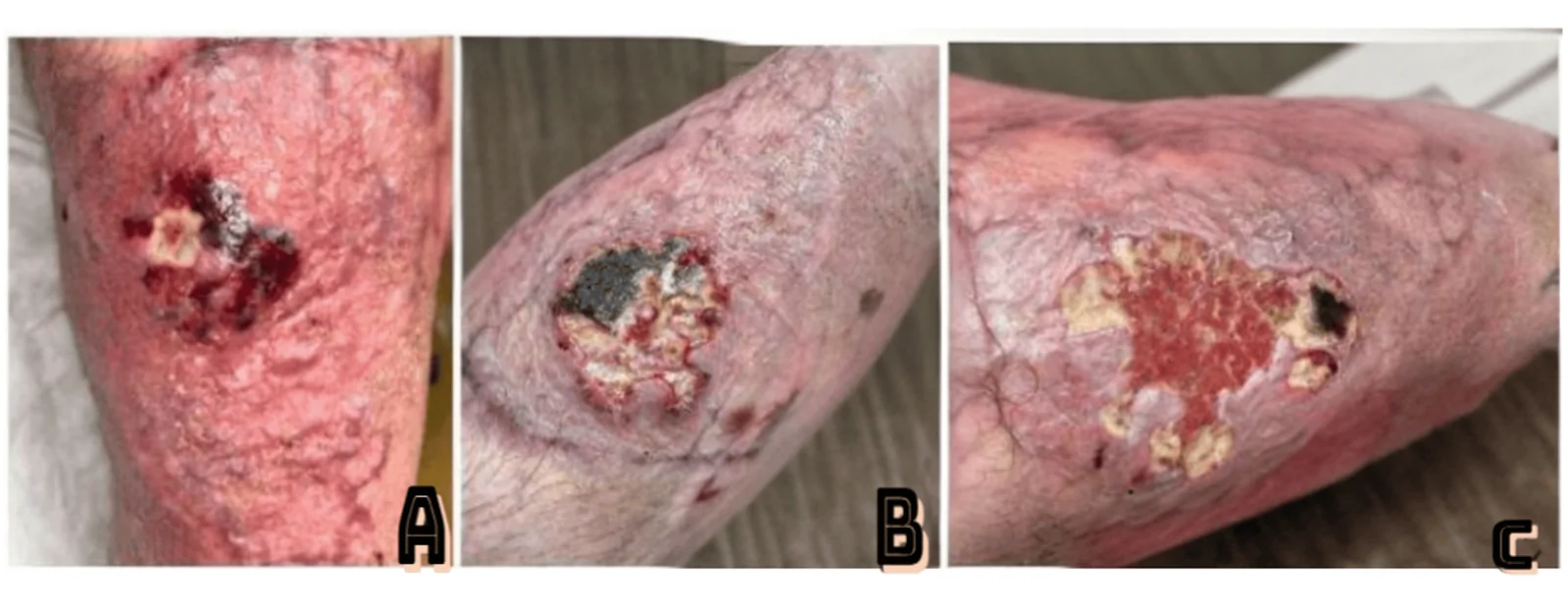
Evolution of wounds after repeated skin grafting (A-C) Posterior lower limb distal to the popliteal space; evolution of a recurrent ulcerative lesion within a prior skin graft; evidence of skin necrosis. A is oriented with the superior aspect of the wound at the top of the photo; B and C have the superior aspect of the wound to the left of the photos

**Figure 5:**
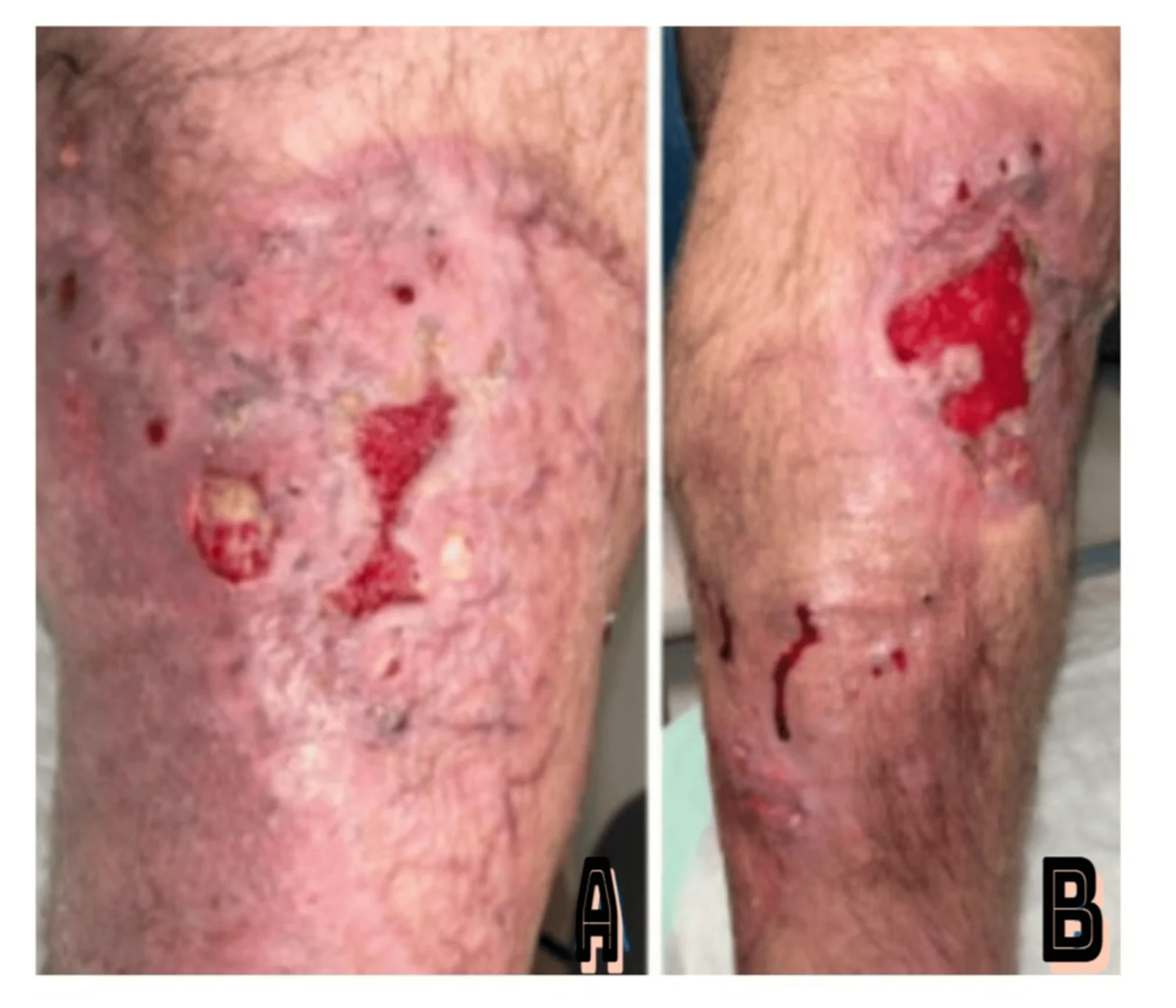
Posterior calf wound (L), anterior tibia wound (R) with local wound care (A-B) Additional ulcerative lesions on the anterior tibia, with the superior aspect of the wounds at the top of the photo

While these methods allowed for some improvement in the wounds as visualized above, the patient unfortunately returned to the hospital with worsening wounds in late spring 2025. The above wounds were noted to have new nodules with seropurulent drainage, and necrotic eschar was visualized on both the anterior tibia and posterior calf (Figure 6). The anterior tibial lesion was excised down to the ulcer bed, and final pathology resulted in the diagnosis of NL.

**Figure 6:**
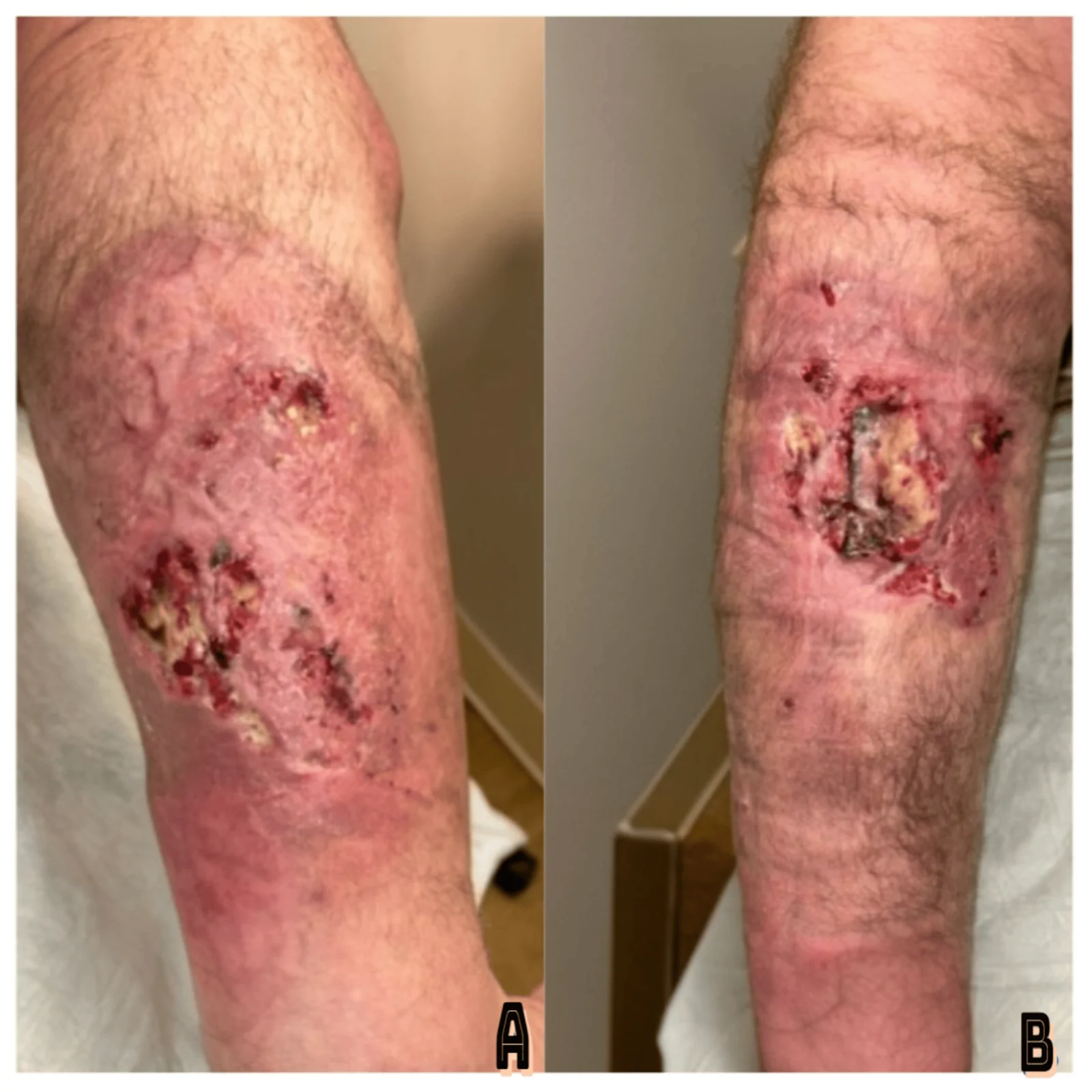
Ulcer progression, posterior calf (L), anterior tibia (R) (A) Additional ulcerative lesions with skin necrosis along the posterior calf, with a proximal wound at the top of the photo. (B) Necrotic ulcerative lesion on the anterior tibia with the proximal aspect of the wound at the top of the photo

The diagnostic specimen slides can be seen below with characteristics of NL such as granulomas as well as the typical findings of layers of necrotic collagen and inflammatory infiltrates within the deep dermis (Figures 7-8). The patient has since been seen by Rheumatology and is now being managed with azathioprine 100 mg daily as well as prednisone 10 mg daily. He has seen a good response with adequate wound healing, as well as a decrease in the number of new lesions present. 

**Figure 7:**
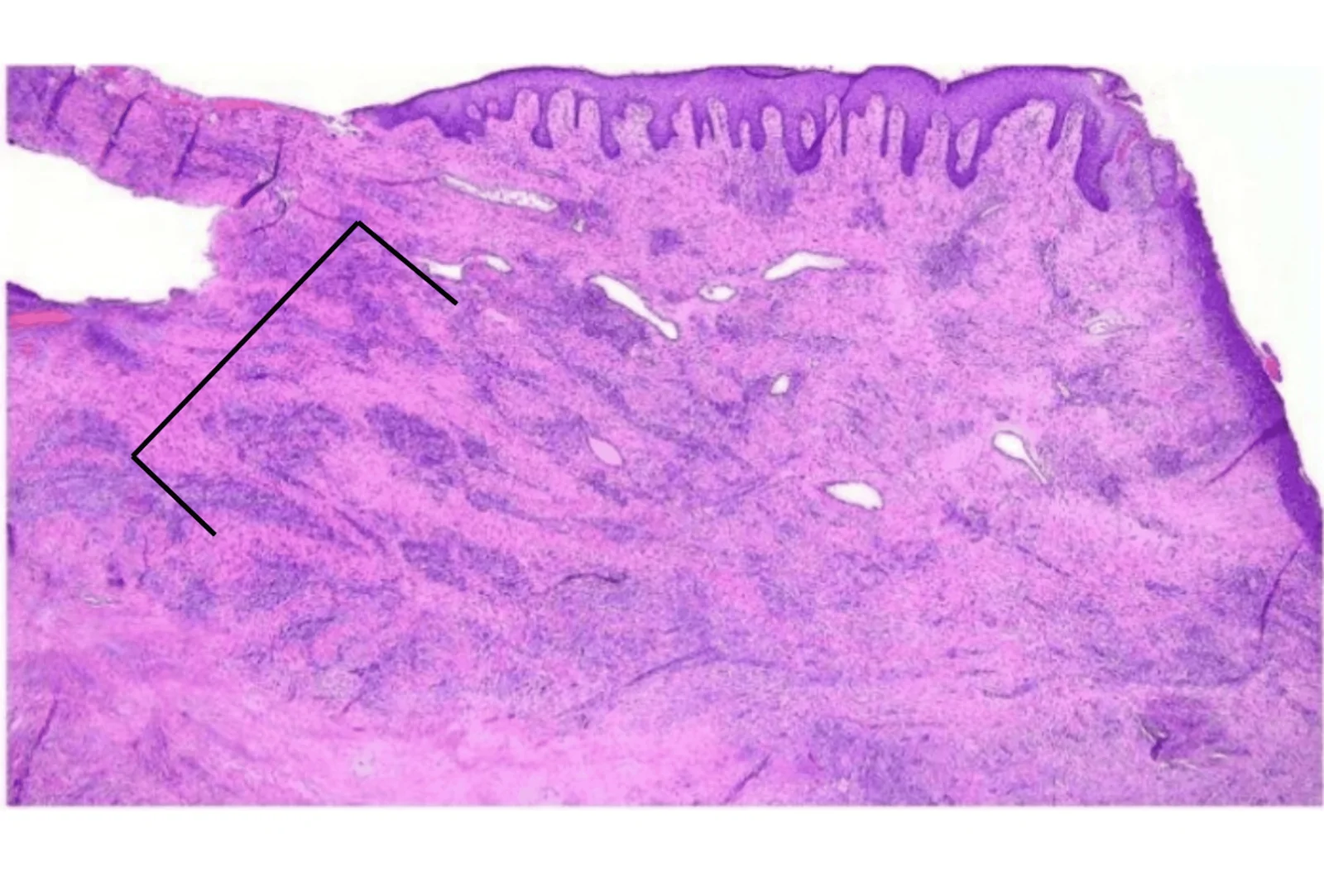
H&E 2x. This image demonstrates the classic "layered cake" (bracket) pattern of necrobiosis lipoidica, alternating necrotic collagen and inflammatory infiltrates involving up to the deep dermis

**Figure 8:**
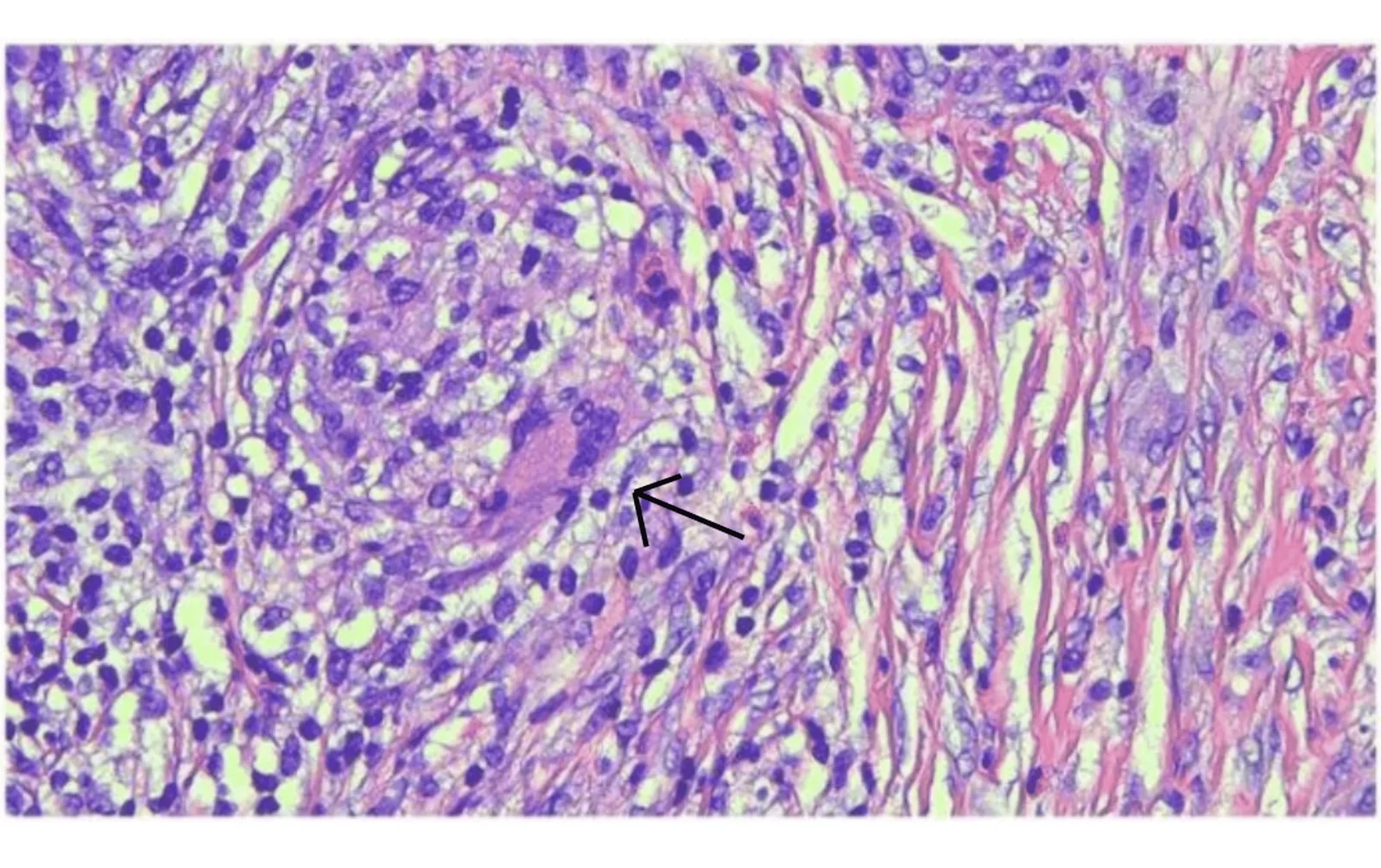
H&E 40x. This image shows a granuloma with a multinucleated giant cell, lymphocytes, occasional eosinophils and plasma cells (arrow). This granuloma is not surrounded by a distinctive ring of lymphocytes (nonpallisading), more common in NL of a nondiabetic patient NL: necrobiosis lipoidica

## Discussion

In the case of our patient, the diagnosis was delayed for nearly two years due to concern for infectious etiologies. Due to this, he underwent several surgical resections in hope of obtaining source control. This unfortunately resulted in increased cost for this patient for ineffective treatments. Additionally, he had to stop doing many of his typical activities and hobbies due to the open wounds as well as the presence of a midline for long-term antibiotics. An earlier diagnosis would have allowed for effective treatment, as well as less costs both financially and psychosocially. 

The diagnosis of NL is dependent upon both clinical and pathophysiologic evaluations. Clinical diagnosis can be determined by pretibial atrophic patches, which can vary in color from yellow-orange, pink-brown, or red-brown. It is recommended that if biopsies are to be taken, they should extend to the subcutaneous fatty layer and are generally more successful with a biopsy at the edge of the lesion. Histology will reveal layers of collagen and inflammatory infiltrates (Figures 7-8) [2]. 

The current treatment recommendations are focused on advancing treatment in a stepwise fashion, starting with topical corticosteroids, and progressing through the treatment algorithm as needed based upon response [2]. However, despite these guidelines, and this disease being identified nearly 100years ago, there is still a general lack of knowledge of how to induce remission. Within the last few years, two systematic reviews have been completed analyzing case studies and case series. 

In 2023, a systematic review performed by a group of dermatologists included 54 case reports, two case series, and four retrospective cohort studies to give a total of 375 patients. This paper found that phototherapy was the most studied treatment, and that topical steroids were the least effective monotherapy. The authors also raise concern about topical corticosteroids leading to further skin atrophy. Among the multiple medical treatments discussed, interestingly, the authors found a study that supported the use of compression stockings to prevent ulcers, as well as improvement of current. This seems to be supportive of the idea that NL is multifactorial in etiology and is associated with vascular abnormalities [4]. 

A 2024 systematic review, which analyzed a total of 19 original studies, drew attention to better control of NL lesions with increased glycemic control for those with diabetes. Curiously, they also discuss the link between arterial hypertension and NL: this is the second most common association after diabetes. Once again, this seems to support the link between vascular abnormalities in the pathophysiology of the disease. [5] 

Between these two systematic reviews, it seems that the best treatment choices start with control of underlying comorbidities, most notably glycemic control. Our patient did not have a history of diabetes, nor any history of autoimmune-mediated diseases. He does not take medications for hypertension. Lab work performed during a few of his hospitalizations found normal thyroid-stimulating hormone (TSH) levels. Therefore, there do not seem to be underlying comorbidities for this patient to control. He has since followed up with rheumatology and now has better control with immunosuppressants (azathioprine, prednisone).

## Conclusions

This study reviewed the case of delayed diagnosis, which ultimately resulted in multiple surgical interventions and a prolonged course of intravenous antibiotics. An earlier accurate biopsy would have assisted in the appropriate diagnosis and treatment. In the future, a multidisciplinary approach should be taken to further research in the diagnostic criteria and treatment guidelines. Surgical intervention should be held as a last resort for necrotic lesions, as this disease is known to have pathergy. 
